# A mixed-methods approach exploring acceptability and feasibility of trials designed to test drugs targeting prevention of post-traumatic osteoarthritis after knee injury

**DOI:** 10.1302/2046-3758.139.BJR-2024-0109

**Published:** 2024-09-19

**Authors:** Raneem Kalsoum, Catherine J. Minns Lowe, Sophie Gilbert, Andrew W. McCaskie, Martyn Snow, Karina Wright, Geoff Bruce, Deborah J. Mason, Fiona E. Watt

**Affiliations:** 1 Department of Immunology & Inflammation, Imperial College London, London, UK; 2 Musculoskeletal Research Unit, School of Health and Social Work, University of Hertfordshire, Hatfield, UK; 3 Biomechanics and Bioengineering Research Centre Versus Arthritis, School of Biosciences, Cardiff University, Cardiff, UK; 4 Division of Trauma and Orthopaedic Surgery, Department of Surgery, Addenbrooke's Hospital, Cambridge, UK; 5 University of Cambridge, Cambridge, UK; 6 Keele University, Keele, UK; 7 The Royal Orthopaedic Hospital, Birmingham, UK; 8 The Robert Jones and Agnes Hunt Orthopaedic Hospital, Oswestry, UK; 9 Centre for Osteoarthritis Pathogenesis Versus Arthritis, Kennedy Institute of Rheumatology, NDORMS, University of Oxford, Oxford, UK; 10 Centre for Sports, Exercise and Osteoarthritis Versus Arthritis, NDORMS, University of Oxford, Oxford, UK; 11 Department of Rheumatology, Imperial College Healthcare NHS Trust, London, UK

**Keywords:** Injury, Knee, Post-traumatic osteoarthritis, post-traumatic osteoarthritis, post-traumatic osteoarthritis, knee joint injuries, clinicians, knee, Osteoarthritis (OA), joint injury, knee symptoms, BMI, clinical trials

## Abstract

**Aims:**

To explore key stakeholder views around feasibility and acceptability of trials seeking to prevent post-traumatic osteoarthritis (PTOA) following knee injury, and provide guidance for next steps in PTOA trial design.

**Methods:**

Healthcare professionals, clinicians, and/or researchers (HCP/Rs) were surveyed, and the data were presented at a congress workshop. A second and related survey was then developed for people with joint damage caused by knee injury and/or osteoarthritis (PJDs), who were approached by a UK Charity newsletter or Oxford involvement registry. Anonymized data were collected and analyzed in Qualtrics.

**Results:**

Survey responses (n = 19 HCP/Rs, 39 PJDs) supported studies testing pharmacological agents preventing PTOA. All HCP/Rs and 30/31 (97%) PJDs supported the development of new treatments that improved or delayed knee symptoms and damage to knee structure. PJDs thought that improving structural knee damage was more important than knee symptoms. Both groups found studies more acceptable as expected future benefit and risk of PTOA increased. All drug delivery routes were acceptable. Workshop participants (around n = 60) reflected survey views. Discussions suggested that stratifying using molecular testing for likely drug response appeared to be more acceptable than using characteristics such as sex, age, and BMI.

**Conclusion:**

Our findings supported PTOA drug intervention studies, including situations where there is low risk of disease, no expected benefit of treatment, and frequent treatment administration. PJDs appeared less risk-averse than HCP/Rs. This work reinforces the benefits of consensus and involvement work in the co-creation of PTOA drug trial design. Involvement of key stakeholders, such as PJDs with different risks of OA and regulatory representatives, are critical for trial design success.

Cite this article: *Bone Joint Res* 2024;13(9):513–524.

## Article focus

- We explored key stakeholder views around how feasible and acceptable different aspects of design of experimental medicine studies and clinical trials seeking to prevent post-traumatic knee osteoarthritis (PTOA) would be.
- We identified areas of agreement, difference, and uncertainty within and between two key stakeholder groups (healthcare professional, clinicians and/or researchers, and those with joint damage caused by knee injury and/or osteoarthritis) using a mixed-methods approach: electronic surveys and facilitated group discussions at an in-person workshop.

## Key messages

- There was overall support in both groups across many design elements of PTOA trials and studies. We note some differences between groups in certain areas that would benefit from further exploration by consensus work to maximize trial success.
- Stakeholders supported the testing of treatments that improved both structural knee damage and knee symptoms, and felt that studies were more acceptable where expected benefit and/or risk of PTOA is higher.
- Involving all relevant stakeholders, including those with knee injury and clinicians in the co-creation and design of PTOA studies, will increase the quality and feasibility of PTOA trial design.

## Strengths and limitations

- We collected and analyzed qualitative and quantitative data using different methods to explore diverse perspectives and compare findings between groups.
- Our approaches included relatively low numbers of survey respondents (who were not demographically diverse), introducing the potential for bias.

## Introduction

Osteoarthritis (OA) is the most common form of arthritis, with global cases on the rise.^1,2^ Despite the pain, disability, and socioeconomic impacts of OA, there is a lack of evidence-based treatments and interventions that can prevent, defer, or lessen the severity of subsequent OA. Approximately 50% of people with significant knee joint injuries develop symptomatic radiographic OA within ten years, referred to as post-traumatic OA (PTOA).^3,4^ Given that we know the timing of the risk event – the injury – this presents an immense clinical opportunity to use an intervention after the injury to try to prevent PTOA. One potential modality with a compelling scientific rationale which could modulate outcome, by modulating the biological response to the injury itself, would be pharmacological.^5^

Research groups have sought to define current challenges associated with the design of clinical trials in people at risk of knee PTOA (i.e. secondary prevention trials), which include appropriate or surrogate trial endpoints measuring OA illness and disease, and the ideal design of trials from a regulatory and clinical standpoint.^6^ A consensus exercise reviewed interventional studies at time of injury, and identified key considerations for conducting and designing PTOA prevention studies.^7^ An Orthopaedic Research Society (ORS) workshop in 2019 also highlighted the substantial opportunities for translation of pre-clinical findings to trials studying new interventions.^8^ One identified priority from these exercises was to focus on feasibility and acceptability of testing interventions by involving key stakeholders, including people with knee injury, OA, and/or PTOA, and their healthcare professionals.

To improve research quality, there is a general call to move from treatment-centred to patient-centred research, involving patients in all research stages including its design, and understanding areas of similarity, difference, and uncertainty in key stakeholder beliefs.^9^ This has been shown to enable co-design of higher-quality trials, maximizing chances of successfully bridging the translational gap, getting it ‘right first time’ to bring new treatments to the clinic.^10^

Quality of trial design is a particularly pertinent consideration for OA, which has had well-documented challenges with its drug trials to date and no successfully licensed disease-modifying drugs, despite many attempts.^11,12^ Prevention trials pose yet more challenges, given the need for an ‘early outcome’ and difficulty showing effect in an insidious, heterogeneous disease.^5^ Trial ‘success’ (and failure) can be impacted in various ways by study design, from difficulty recruiting sufficient participants (leading to trial failure), to fidelity issues during the trial (such as use of other treatments), the accurate and reliable detection of its outcome (meaningful to the person and illness), to issues with successful licensing with available trial evidence. ‘Success’ is understandably often seen as the licensing of a new drug for a new indication, which is important commercially and for those living with the disease. A new treatment that modifies progression was a top research priority for common musculoskeletal conditions including OA and joint injury,^13^ but this goes hand-in-hand with a successfully designed and delivered trial.

With the aim of informing how we conceive and design successful prevention trials of PTOA, we set out to explore in a structured way the views and considerations around feasibility and acceptability of different aspects of their design and delivery, including potential barriers. We engaged two key stakeholder groups about experimental medicine studies^14^ and full clinical trials of pharmacological agents in this area: those with joint damage caused by knee injury and/or osteoarthritis (PJDs); and healthcare professionals, clinicians, and/or researchers (HCP/Rs).

## Methods

**Fig. 1:**
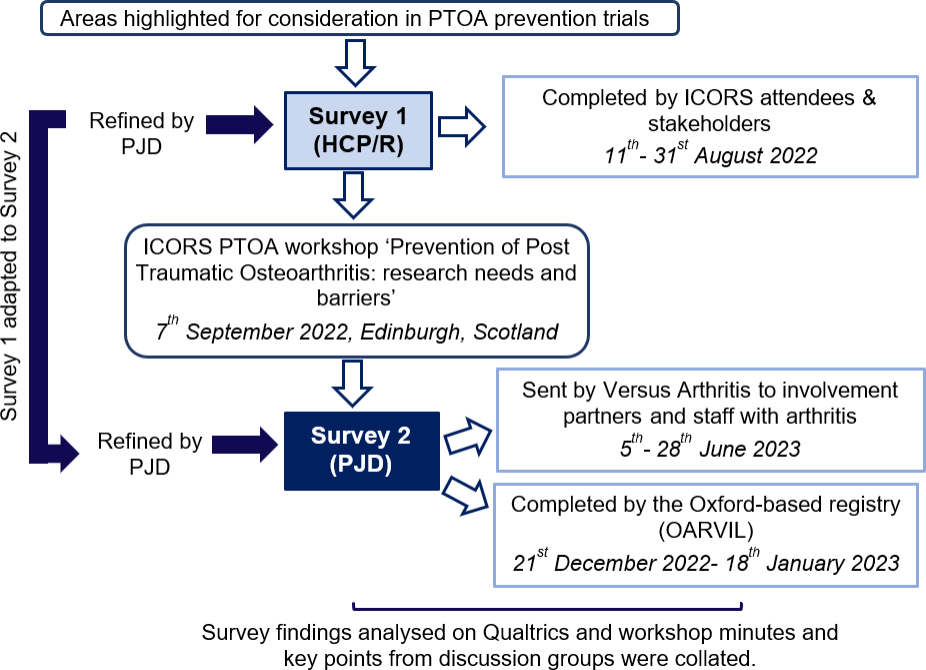
A workflow outlining our mixed-methods approach. HCP/Rs, healthcare professionals, clinicians, and/or researchers; ICORS, International Combined Orthopaedic Research Societies; OARVIL, Osteoarthritis Research Voluntary Interested List; PJDs, people with joint damage caused by knee injury and/or osteoarthritis; PTOA, post-traumatic osteoarthritis.

We employed two parallel, related processes in a mixed-methods approach. Data were collected in an initial survey (Survey 1) aimed at HCP/Rs, ahead of an interactive workshop, where further data were collected using facilitated face-to-face discussion groups. A second survey (Survey 2), derived from Survey 1 and aimed at self-reporting PJDs, was undertaken following the workshop (Figure 1).

### Facilitated workshop

A workshop proposal (DJM, FW) was accepted for the International Combined Orthopaedic Research Societies (ICORS) 2022, a triennial multi-society meeting attended by over 500 participants and co-hosted by the British Orthopaedic Research Society (BORS), the European Orthopaedic Research Society (EORS), and the AO Research Institute Davos. The workshop, entitled “Prevention of Post-Traumatic Osteoarthritis: research needs and barriers”, was held on 7 September 2022 in Edinburgh, UK. Attendees comprised clinicians, engineers, clinical researchers, and scientists. Workshop information was circulated to meeting registrants by ICORS organizers prior to the workshop.

Two patient representatives were invited to the workshop via an Oxford registry and the Involvement team at Versus Arthritis, a UK-based musculoskeletal charity. Representatives were provided with a lay information sheet prior to attending the workshop and invited to ask questions about their contribution before agreeing to participate.

The workshop programme included a keynote talk on the topic. Survey 1 summary results were presented to workshop attendees to provide context (Supplementary Figure a). Three discussion groups (15 to 20 individuals/group) were held with pre-appointed facilitators who had knowledge of the area and experience of facilitation. They ensured that discussions were focused, conversations were aligned, and everyone was heard, valued, and respected.^15^ Groups were intentionally interdisciplinary with two groups including patient representatives. Facilitators were briefed and provided with ‘prompt questions’ (Supplementary Tables i to iii) for each of the following groups prior to the workshop: ‘How do we design studies/trials that are feasible?’ (Group A, facilitated by AM and SG); ‘How do we design studies/trials that are acceptable?’ (Group B, facilitated by MS and DJM); ‘What should experimental medicine studies consider?’ (Group C, facilitated by FW and KW).

### Workshop data collection and reporting

The group’s chosen reporter presented their group’s discussion to all attendees during the final session at the workshop. Written notes were compiled by the reporter, and the subsequent minutes were reviewed and approved by all facilitators and used as source data for this article.

### Survey development and delivery

Six areas where we wanted to assess perceptions/beliefs were selected as survey domains (from previously identified important considerations for trial design following knee injury):^7^ 1) Testing current and new treatments; 2) Symptoms versus structure; 3) Risk versus benefit (included changing risks of PTOA for an individual, drug safety, and how a drug works); 4) How a drug might be taken; 5) Frequency of drug administration; and 6) Timing of intervention.

### Pre-workshop Survey 1, for HCP/Rs

Survey 1 was developed, iteratively designed, and reviewed by FW and DJM (full text of Survey 1, Supplementary Material 2). It was further reviewed and refined by a patient representative with knee OA, who also piloted the survey prior to its distribution. The link to Survey 1 was emailed to registered attendees of the workshop by meeting organizers one month prior to the workshop, and was live between 11 and 31 August 2022.

### Post-workshop Survey 2, for PJDs

RK, FW, and DJM adapted Survey 1 to Survey 2 using understandable language for PJDs who were aged 18 years and above. A significant knee injury was defined by the individual’s inability to put their full weight through the knee for at least 48 hours following an injury episode, and clinician assessment and/or MRI showing that they had injured a structure within the knee. The same domains and question types were retained for results from the two surveys to be compared. The text was further reviewed by a patient representative with past knee injury (GB) and the registry coordinator (Full text of Survey 2, Supplementary Material 3). An invitation with an explanation and link to Survey 2 was sent to members of the Oxford-based registry ‘Osteoarthritis Research Voluntary Interested List’ (OARVIL), who had knee OA and previously indicated willingness to provide opinions on OA research (survey was live 21 December 2022 to 18 January 2023). Versus Arthritis also emailed the survey to individuals who receive their involvement communications, and to their network of staff with arthritis (survey was live 5 to 28 June 2023) (Figure 1).

### Survey data collection and analysis

Surveys were designed by RK on Qualtrics, an online survey platform hosted by Imperial College London (Qualtrics, USA), with data protection agreements in place. All questions were optional, respondents were able to skip questions they did not wish to answer, and had the option to select ‘not sure’.

All provided data were analyzed and described (i.e. incomplete responses were not excluded). Each survey was reported separately, and findings compared descriptively. Results were analyzed and presented using Qualtrics XM and Excel (Microsoft, USA).

## Results

### Workshop

Around 60 congress attendees attended the workshop, including two patient representatives. For General Data Protection Regulation (GDPR) reasons, their characteristics could not be recorded. Some HCP/R attendees informally reported that they had lived experience of knee injury and/or knee OA. Key discussion points raised by each discussion group are summarized in Supplementary Tables i to iii. A summary of group discussions was presented to all workshop attendees, including points of agreement in the final plenary workshop session (Table I).

**Table I.** Summary of key points from the workshop.

Area	Key points	Supporting points
Overarching	Future PTOA trials are needed and supported	Early treatments that seek to prevent PTOA (i.e. around the time of injury) are likely to be feasible and acceptable with the right processes in place
Design of trials	It is important to have those who have experienced a knee injury, those with PTOA, and clinicians delivering care involved in trial design	
	Approaches to stratification are supported in principle, but there are barriers to this that need to be addressed	The method of selecting those at ‘high’ and ‘low’ risk at the time of injury needs careful consideration
		Stratification using molecular data was seen as more acceptable than stratification based on demographic features such as sex, age, or modifiable factors like BMI
		The likely effect of the specific treatment target should be considered
		How we screen/enrol people for trials should be based on the person’s likelihood to respond to that treatment
		More work is needed to decide the choice and best use of preclinical models and human studies that establish molecular predictors of outcome, including mechanistic readouts of target response
New target development for trials	Improved understanding of disease pathology is supported to identify novel targets and enhance design of experimental medicine studies	Targeting symptoms as well as prevention of structural PTOA would be likely to increase appeal to younger patients

PTOA, post-traumatic osteoarthritis.

### Surveys

Characteristics of respondents based on the optional demographic information they provided are summarized in Table II.

**Table II.** Characteristics and survey completeness in survey respondent groups.

Demographic categories	Survey 1 (HCP/R), n (%)		Survey 2 (PJD), n (%)
**Survey access and completeness**			
Total access	n = 30		n = 46
Fully completed	15/30 (50)		29/46 (63)
Partially completed	4/30 (13)		10/46 (22)
Accessed, but not completed	11/30 (37)		3/46 (7)
Did not fulfil the inclusion criteria	0 (0)		4/46 (9)
**Ethnicity**			
White	8/13 (62)		26/27 (96)
Asian or Asian-British	3/13 (23)		0 (0)
Other	2/13 (15)		1/27 (4)
**Gender identity**			
Female	6/14 (43)		21/28 (75)
Male	8/14 (57)		7/28 (25)
**Age, yrs**			
18 to 29	5/14 (36)		0 (0)
30 to 39	3/14 (21)		0 (0)
40 to 49	2/14 (14)		4/28 (14)
50 to 59	2/14 (14)		4/28 (14)
60 to 69	2/14 (14)		14/28 (50)
70 to 79	0 (0)		3/28 (11)
80 to 89	0 (0)		3/28 (11)
**Respondent category (Survey 1)**		**Respondent category (Survey 2)**	
Clinicians	3/18 (17)	Knee OA	17/30 (57)
Researchers (non-clinicians)	11/18 (61)	Knee arthroplasty due to previous knee OA	7/30 (23)
Orthopaedic surgeons	2/18 (11)	Current knee injury	2/30 (7)
Other healthcare professionals	1/18 (6)	Knee OA and past knee injury	4/30 (14)
Other	1/18 (6)		

Completeness was described as: Full (fully completed, i.e. where all questions were answered); Partial (partially completed, i.e. where some questions were answered), and Access only (where individuals accessed and reviewed the entire survey but did not answer any questions).

HCP/Rs, healthcare professionals, clinicians, and/or researchers; N/A, not applicable; OA, osteoarthritis; PJDs, people with joint damage caused by knee injury and/or osteoarthritis.

**Fig. 2:**
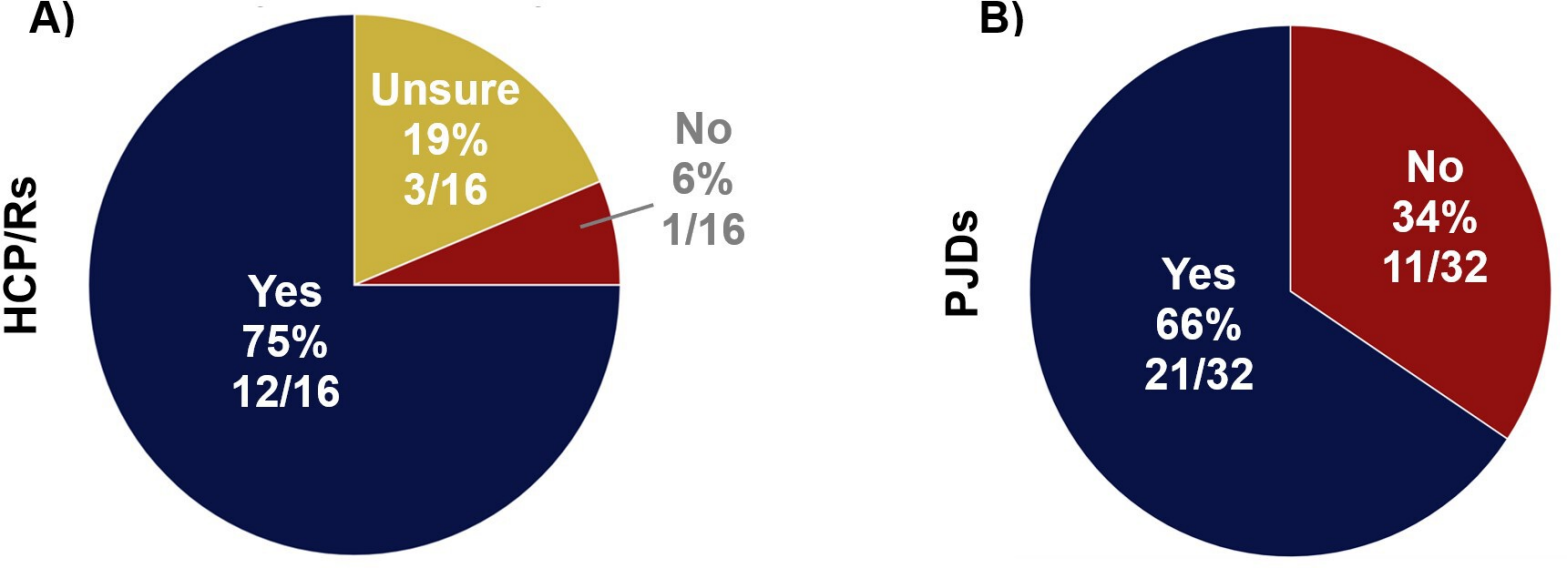
Findings from surveys when respondents were asked whether they knew the difference between a clinical trial, an experimental medicine study, and a feasibility study. Survey findings for: a) healthcare professionals, clinicians, and/or researchers (HCP/Rs) from Survey 1; and b) people with joint damage caused by knee injury and/or osteoarthritis (PJDs) from Survey 2.

Most respondents knew the difference between a clinical trial, an experimental medicine study, and a feasibility study (HCP/Rs 12/16 (75%) and PJDs 21/32 (66%)) (Figure 2).

**Fig. 3:**
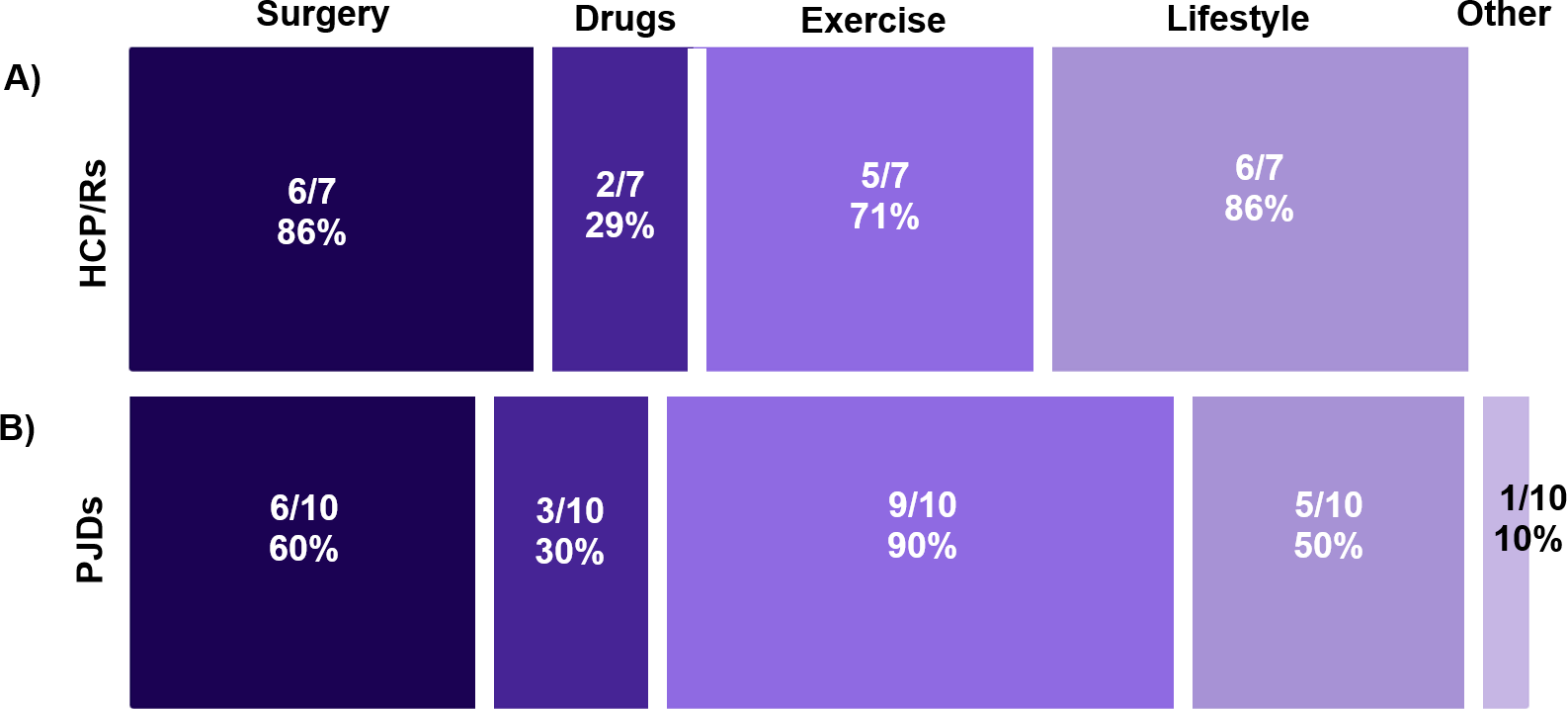
Findings from surveys when respondents were asked which treatments they thought were effective in preventing post-traumatic osteoarthritis (PTOA). Survey findings for: a) healthcare professionals, clinicians, and/or researchers (HCP/Rs) from Survey 1 and b) people with joint damage caused by knee injury and/or osteoarthritis (PJDs) from Survey 2. Respondents who felt there were effective treatments for preventing PTOA were asked which one of five treatment categories they considered effective. Multiple answers could be selected. Overall, 7/14 HCP/R respondents gave a total of 19 responses and 10/29 PJD respondents gave a total of 24 responses.

Respondents to both surveys supported the testing of existing treatments seeking to prevent PTOA in human clinical trials (PJDs 29/31 (83%)) (HCP/Rs 15/18 (83%)). However, many PJDs (12/26 (46%)) were unsure whether we have current effective treatments in preventing PTOA development, and most HCP/Rs (13/19 (68%)) thought we did not. Considering only the respondents who indicated that there was current effective treatment in preventing the development of PTOA, PJDs felt that exercise was the most effective (9/10 (90%)), whereas HCP/Rs thought this was lifestyle modification (6/7 (86%)) and surgery (6/7 (86%)) (Figure 3).

All HCP/Rs and most PJDs (30/31 (97%)) supported the development of new treatments that improve or slow down knee symptoms and damage to the knee structure, rather than targeting either symptoms or structure alone. Overall, 24/32 (75%) PJDs thought that slowing down damage to the knee structure was more important than improving or slowing down knee symptoms. This information was introduced to Survey 2, and was therefore missing for HCP/Rs.

No PJD found it unacceptable to test new drugs (which had unknown/no effect on knee symptoms) in a clinical trial or experimental medicine setting that sought to improve symptoms and/or joint damage. However, some HCP/Rs thought that it was unacceptable to administer a drug which improves knee symptoms with no effect on joint damage (3/14 (21%)), or one that slows/improves joint damage with no effect on knee symptoms (3/14 (21%)).

**Fig. 4:**
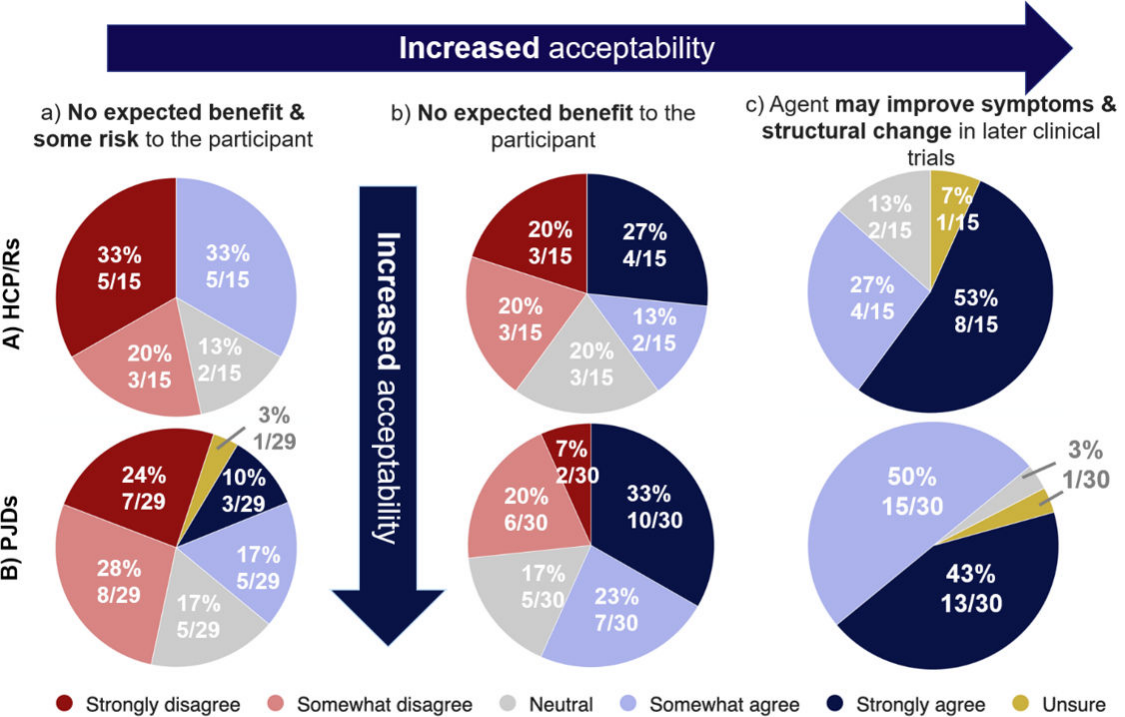
Findings from surveys when respondents were asked about how acceptable they believed testing agents in human experimental medicine studies in scenarios a, b, and c to be. Survey findings for: a) healthcare professionals, clinicians, and/or researchers (HCP/Rs) from Survey 1; and b) people with joint damage caused by knee injury and/or osteoarthritis (PJDs) from Survey 2. The six Likert scale options are listed in the key.

Both groups found it more acceptable to test agents in human experimental medicine studies, as the expected benefit to the participant increased (Figure 4). A total of 12/15 (80%) HCP/Rs and 28/30 (93%) PJDs agreed with testing agents that may improve symptoms and structural change in later clinical trials, compared to 5/15 (33%) HCP/Rs and 8/29 (27%) PJDs who agreed with testing agents that had no expected benefit and some risk to the participant (i.e. the situation in an experimental medicine study). More PJDs agreed with or were neutral about testing agents with more challenging risk-benefit profiles compared with HCP/Rs: 6/15 (40%) HCP/Rs, but only 8/30 (27%) PJDs found it unacceptable to test treatments with no expected benefit.

All target groups for PJDs and most for HCP/Rs were acceptable groups to offer experimental medicine studies seeking to test new interventions (Supplementary Figure b). In total, 17/30 (57%) PJDs and 7/15 (47%) HCP/Rs felt that people who had a clinically significant knee injury were an acceptable target group. Fewer PJDs (8/30 (27%)) and HCP/Rs (3/15 (20%)) found healthy individuals without knee injury or OA an acceptable group.

**Fig. 5:**
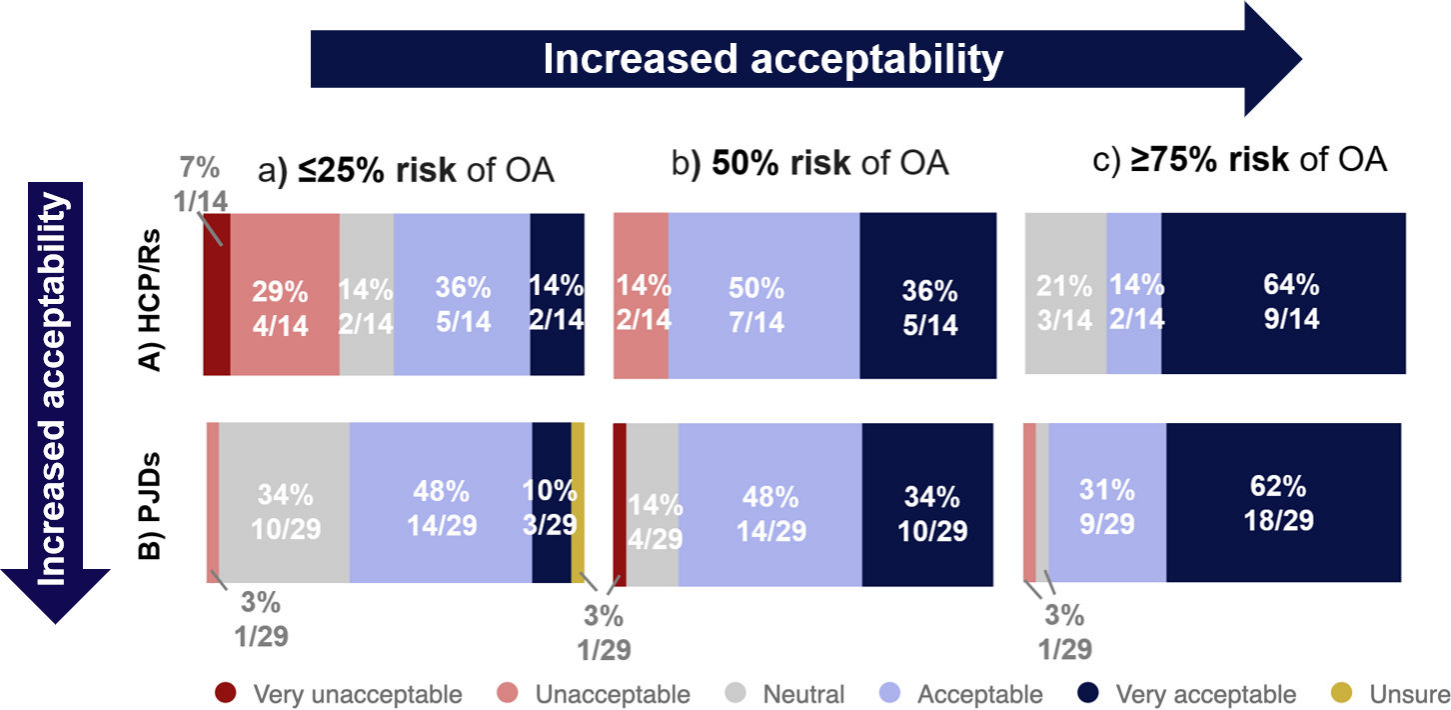
Findings from surveys when respondents were asked about how acceptable they thought it was to test a new drug in a clinical study that seeks to prevent post-traumatic osteoarthritis (PTOA) in people with different risks of OA. Survey findings for: a) healthcare professionals, clinicians, and/or researchers (HCP/Rs) from Survey 1; and b) people with joint damage caused by knee injury and/or osteoarthritis (PJDs) from Survey 2. Six Likert scale options are listed in the key.

Acceptability to test new drugs in a clinical study increased as the risk of developing PTOA increased (Figure 5). Acceptability for HCP/Rs increased from 7/14 (50%), where there was a ≤ 25% risk of OA, to 11/14 (78%), where there was a ≥ 75% risk of OA (with those remaining being neutral). A similar trend in acceptability was observed in PJDs: 17/29 (58%), where there was a ≤ 25% risk of OA, compared to 27/29 (78%), where there was a ≥ 75% risk of OA. Overall, a higher proportion of PJDs compared to HCP/Rs found it acceptable or were neutral about testing new drugs across groups at different risk for OA.

There was consensus between both HCP/Rs and PJDs on the most acceptable target groups for testing a new, well-tolerated, and reasonably safe drug in a clinical study that sought to prevent PTOA (Supplementary Figure c). Individuals with knee OA were the most acceptable, while those where the drug worked in other conditions but was not yet tested in OA (repurposed) were the least acceptable.

**Fig. 6:**
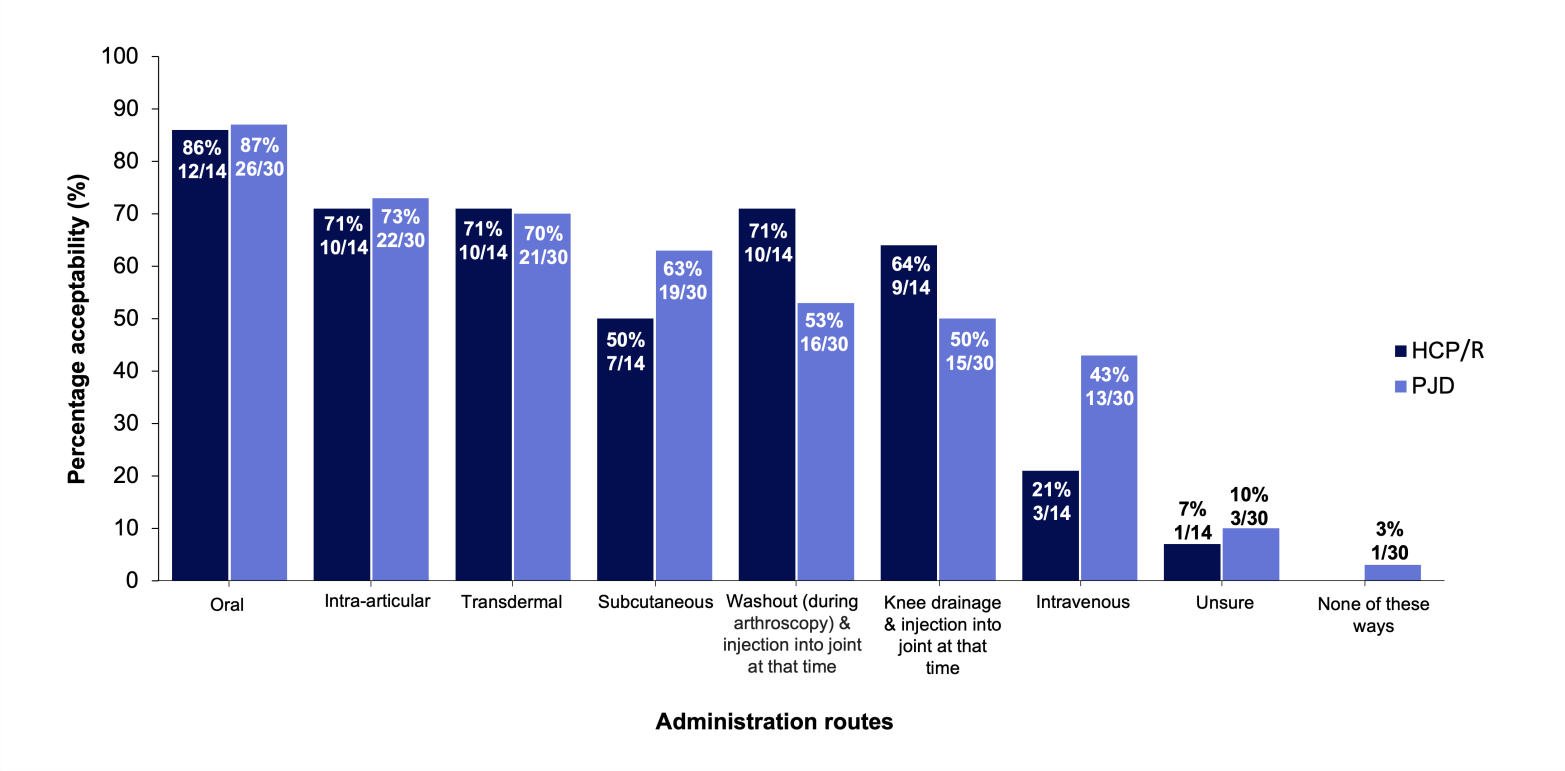
Findings from surveys when respondents were asked about how acceptable they thought different drug administration routes are. a) Healthcare professionals, clinicians and/or researchers (HCP/R) answers from Survey 1. b) People with joint damage caused by knee injury and/or osteoarthritis (PJD) answers from Survey 2. Multiple answers could be selected; 14 HCP/R respondents gave a total of 41 responses, and 30 PJD respondents gave a total of 136 responses.

HCP/Rs and PJDs were asked to select all drug administration routes they deemed as acceptable (Figure 6). All delivery routes were acceptable, except for one PJD who suggested that none were acceptable. Oral intake was the most acceptable route (26/30 (87%) PJDs and 12/14 (86%) HCP/Rs). Both groups suggested similar acceptability for intra-articular injection (22/30 (73%) PJDs and 10/14 (71%) HCP/Rs) and transdermal route (21/30 (70%) PJDs and 10/14 (71%) HCP/Rs). These were more acceptable than systemic injections (13/30 (43%) PJDs and 3/14 (21%) HCP/Rs).

HCP/Rs were less accepting of more frequent drug administration than PJDs (Supplementary Figure d). A total of 25/28 (89%) PJDs thought that single injections within four to six weeks of injury, monthly injections over three months, and three-monthly injections over one year were acceptable, compared with a similar proportion of HCP/Rs (12/14 (86%)) for all these options. Overall, 25/28 (89%) PJDs thought that weekly injections over two to four weeks were acceptable, but fewer HCP/Rs (7/14 (50%)) agreed with this; 23/28 (82%) PJDs and 11/14 (79%) HCP/Rs found single injections within two weeks of injury acceptable.

## Discussion

This study found that two key stakeholder groups showed high overall acceptance and support for testing pharmacological agents seeking to prevent knee PTOA, with more points of agreement than difference or uncertainty. It was of note that there was no complete opposition to any design element, with options of ‘very unacceptable’ or ‘strongly disagree’ rarely selected by respondents.

A consistent finding that emerged from both groups was a belief in the lack of current effective treatments for preventing the development of PTOA, and overall support for testing new treatments in clinical trials and experimental medicine studies. In the workshop discussions, the need for preclinical evidence and acceptable safety profiles to support testing new and existing agents in humans was emphasized.

This study also provides us with useful information that will contribute to how we design and deliver high-quality trials in this area, and we highlight what we consider to be key findings here. Both groups agreed that developing treatments that improved structural knee damage and knee symptoms was important rather than targeting either issue alone. PJDs thought that improving structural knee damage was more important than knee symptoms (HCP/Rs were not asked this question). This affirms findings from previous exercises which showed that both symptoms and structural damage were felt to be important, with a recommendation that they should both be measured in such trials.^6,7^ Ongoing efforts have defined PTOA as both a disease (pathophysiology measured with molecular/structural outcomes) and an illness (relating to the experience of individuals and measured as symptoms including pain and reduced quality of life).^16^ Given that there is no current cure for OA disease, focus has been on providing effective symptomatic treatments for ‘OA illness’ in randomized controlled trials (RCTs).^17^ This is supported from a regulatory standpoint, where a clinical benefit needs to refer to a clinically relevant and meaningful change in how a patient feels, functions, or survives.^18^ It is important to note that our findings from PJDs conflict with this approach, suggesting the need to focus on structural protection over symptoms. Workshop discussions also highlighted the need to better understand the underlying pathogenesis of PTOA for the development of experimental design and identification of novel targets. Additionally, given that a high proportion of knee OA cases are asymptomatic during the early stages of the disease,^19^ structure-focused approaches in PTOA trial design and development may be more relevant.

The balance of benefit versus risk to the participant of developing knee OA appeared important when considering how acceptable testing agents were in human experimental medicine studies. Overall, HCP/Rs were more conservative in their views than PJDs. However, more than half of HCP/Rs and PJDs still found clinical trials testing new treatments acceptable when the risk of OA was < 25%, and over 80% approved when OA risk increased to > 50%. The notion that studies need to identify and include ‘high-risk’ patients, and the importance of assessing individual risk (but also likely individual benefit),^20^ was reinforced by both groups. Workshop discussions also supported risk stratification in trial enrolment, with a focus on those at highest risk or with a relevant identifiable disease phenotype. Highlighted considerations included how this risk measurement should be achieved in practice and whether applying a ‘one size’ risk assessment to all trials is feasible. Another accepted view from discussion groups was that stratification approaches should be tailored to identifying a defined group with high likelihood of a detectable clinically significant response to that treatment. The acceptability of stratifying by known risk factors for knee OA using patient characteristics such as sex, age, or BMI was limited, with a preference instead for using molecular tests to achieve stratification. Further work on assessment of personalized risk-benefit, and methods for achieving this, will be critical in understanding what is acceptable across stakeholder groups. In particular, and as addressed in workshop discussions, accurate prediction models and identification of early biomarkers to identify those at higher risk are needed. Regulators rely on accumulated risk-benefit evidence to justify a label of ‘disease modifying’.^21^ The consensus, as well as differing views, between stakeholder groups when considering risk-benefit, as revealed by our study, will be a critical consideration in obtaining a ‘prevention label’ for PTOA.

Both groups felt that there were different current treatments effective in preventing the development of PTOA. Recent efforts have identified guidelines for treatment decisions.^22^ As not all those with knee injury receive or benefit from one type of intervention only,^23^ further investigation of stakeholder acceptability of combination therapies in different target populations, and establishing what would be perceived as ‘usual care’ (for comparator/control arm design), is important but beyond the scope of this current study.

All drug administration routes were acceptable and feasible to both groups, although the workshop specified that a scientifically valid reason for the route should be proposed. Both groups found oral administration the most acceptable, consistent with studies showing that people with OA prefer oral medications.^24^ Interestingly, differences between preferences for oral administration and intra-articular injection, either within or between the stakeholder groups, were small. In support of this, workshop discussions suggested that local intra-articular injections may be more attractive than oral treatments as they minimize systemic side effects and off-target effects compared with systemic administration. These findings provide valuable information as to which routes are most acceptable for both groups, and imply likely adherence and tolerability in PJDs. Finding the optimal balance in administration route acceptability between the HCP/Rs administering the drug, and PJDs receiving it, is critical for trial recruitment and retention, as well as successful implementation into clinical care. Addressing further considerations of differing drug delivery routes (and in other stakeholders such as pharmaceutical companies), including potential and perceived adverse events, duration of action, relative efficacy, and cost-effectiveness/commercial considerations, would expand these findings further.

An important overarching finding was that PJDs were less risk-averse than HCP/Rs to certain trial design elements. PJDs were more accepting of interventions in those with a lower risk of OA, when there was less expected benefit or of increased drug administration frequencies, compared with HCP/Rs. Frequency of drug administration has been reported as a lesser contributing factor for patients when choosing OA medicine, compared with other factors such as risk of side effects, addiction, or availability.^24^ It may be that a higher frequency of clinician-delivered treatment, such as intra-articular injection, was a greater concern for HCP/Rs, given the challenges of limited resources and repeat timed appointments (and awareness of these cost implications).^25^ Alternatively, clinicians might have considered tolerability or safety aspects of repeated invasive procedures differently, although this would need to be explored further. A greater proportion of PJDs than HCP/Rs strongly agreed with testing agents where there was no expected benefit and some risk to the participant (as is seen in, for example, experimental medicine studies or early phase trials). However, a larger proportion were also uncertain. As uncertainty could affect participation, it is critical for additional involvement work to explore the extent and reasons for these uncertainties.

This work has also highlighted the need for greater understanding of the ‘ideal’ primary outcome measures for these trials, the effects of differing risk profiles on decision-making by the patient or clinician (e.g. risk of OA, risk of side effects), and the acceptability of differing methodological approaches to stratification. International interdisciplinary efforts will help to refine approaches to PTOA trial design in this growing area of research interest and activity.^6^ None of these areas would appear ‘un-solvable’, and while further research (including exploration of existing data) is supported, the immense clinical and commercial opportunity presented here should not be underestimated, not least given the possibility for patient benefit and insights for OA as a whole.

There were some limitations to the approaches we used. There was a relatively low total number of respondents in both surveys despite efforts to publicize them. This increased the possibility of unrepresentative findings that cannot be generalized. Although there was potential for bias within the small discussion groups, some findings were also reflected in surveys. Survey 2 had more respondents than Survey 1, which could lead to imbalance in conclusions. Sex, age, and ethnicity also differed between survey groups: Survey 2 respondents were older than a typical knee-injured population (e.g. a median age of 26 years in one cohort study of knee-injured individuals).^26^ Furthermore, we had under-representation from knee-injured people without OA, which should be borne in mind in terms of the generalizability of our findings, but also further work in this area. An insight from this work was the need to establish networks to connect those who have experienced a knee injury into involvement in research. We are not aware of any registries or networks – at least in the UK – seeking to do this.

Survey 1 was aimed at ICORS congress attendees, and specifically people choosing to attend this workshop, which could have introduced confirmation bias (i.e. getting responses from those already motivated by this area). Choosing an orthopaedic congress and interacting with those showing interest by opting for a workshop was intentionally part of our design. HCP/Rs supporting orthopaedic treatment pathways arguably represent those with the most contact (and therefore clinical expertise/key opinion leaders) in treating individuals with PTOA, in collaboration with whom these trials would be run. However, they do not necessarily have specialist expertise or interest in testing or intervening with drugs, i.e. the primary topic of this work. Pre-appointed facilitators were pivotal in ensuring a transparent and inclusive discussion group process. However, these individuals could have been prone to certain biases due to their own expertise.

To maximize diversity in Survey 2 respondents, we included two different recruitment sources. Appropriate electronic devices and emails were necessary to access the survey links, which would have been a barrier to diversity and participation of some underserved groups. We considered, but did not have the resources, to offer paper versions of the surveys, other means of advertising, or translation of the survey into languages other than English.

While some diversity of views was expected and apparent, both stakeholder groups endorsed future PTOA trial design and development as a promising area. These findings provide a nidus of evidence for the first time around the perspectives of relevant stakeholders on what an acceptable, feasible design of drug trials seeking to prevent PTOA after knee injury might look like. However, where there was ‘acceptability’, we note that this was reduced in certain situations (e.g. lower risk of disease, no expected benefit of treatment, and what could be seen as clinically less acceptable treatment administration routes or frequencies). While clinicians were more risk-averse, we found more points of agreement than difference between the two groups.

Although it may not be surprising for key stakeholders to support the ultimate goal of evidence-based interventions that reduce PTOA as a ‘destination’, our findings show their support for the ‘journey’ to get there, by generating high-quality trial data at all stages. Refining trial elements where agreement was shown would seem likely to enhance trial design success and the likelihood of commercially viable trials. We should also continue to communicate and better understand points of divergence, and how these can be narrowed for further synergy.

Based on these data, we would encourage triallists to include PJDs in all stages of their research.^9^ Co-creation frameworks,^27^ Nominal Group Technique, and Delphi studies^28^ are approaches which could further develop these findings and inform trial design in this area. Future conversations and consensus work should include knee-injured people with discernibly different risks of developing PTOA and those who work in industry or regulatory authorities, as well as treating clinicians. Overall, our findings show how seeking and summarizing stakeholder views and perceptions can be used to constructively develop the design of high-quality future drug trials aiming to prevent knee PTOA.

## Data Availability

The datasets generated and analyzed in the current study are not publicly available due to data protection regulations. Access to data is limited to the researchers who have obtained permission for data processing. Further inquiries can be made to the corresponding author.

## References

[1] Long H Liu Q Yin H et al. Prevalence trends of site-specific osteoarthritis from 1990 to 2019: findings from the global burden of disease study 2019 Arthritis Rheumatol 2022 74 7 1172 1183 10.1002/art.42089 35233975 PMC9543105

[2] Yu D Jordan KP Bedson J et al. Population trends in the incidence and initial management of osteoarthritis: age-period-cohort analysis of the Clinical Practice Research Datalink, 1992-2013 Rheumatology (Oxford) 2017 56 11 1902 1917 10.1093/rheumatology/kex270 28977564 PMC5850125

[3] Lohmander LS Englund PM Dahl LL Roos EM The long-term consequence of anterior cruciate ligament and meniscus injuries: osteoarthritis Am J Sports Med 2007 35 10 1756 1769 10.1177/0363546507307396 17761605

[4] Watt FE Gulati M New Drug Treatments for Osteoarthritis: What is on the Horizon? Eur Med J Rheumatol 2017 2 1 50 58 30364878 PMC6198938

[5] Watt FE Posttraumatic osteoarthritis: what have we learned to advance osteoarthritis? Curr Opin Rheumatol 2021 33 1 74 83 10.1097/BOR.0000000000000760 33186246

[6] Whittaker JL Kalsoum R Bilzon J et al. Toward designing human intervention studies to prevent osteoarthritis after knee injury: a report from an interdisciplinary OARSI 2023 workshop Osteoarthr Cartil Open 2024 6 2 100449 10.1016/j.ocarto.2024.100449 38440780 PMC10910316

[7] Watt FE Corp N Kingsbury SR et al. Towards prevention of post-traumatic osteoarthritis: report from an international expert working group on considerations for the design and conduct of interventional studies following acute knee injury Osteoarthritis Cartilage 2019 27 1 23 33 10.1016/j.joca.2018.08.001 30125638 PMC6323612

[8] Mason D Englund M Watt FE Prevention of posttraumatic osteoarthritis at the time of injury: Where are we now, and where are we going? J Orthop Res 2021 39 6 1152 1163 10.1002/jor.24982 33458863

[9] Lacombe D O’Morain C Casadei B et al. Moving forward from drug-centred to patient-centred research: a white paper initiated by EORTC and developed together with the BioMed Alliance members Eur Respir J 2019 53 2 1801870 10.1183/13993003.01870-2018 30792235

[10] Moher D Glasziou P Chalmers I et al. Increasing value and reducing waste in biomedical research: who’s listening? Lancet 2016 387 10027 1573 1586 10.1016/S0140-6736(15)00307-4 26423180

[11] Englund M Osteoarthritis, part of life or a curable disease? A bird’s-eye view J Intern Med 2023 293 6 681 693 10.1111/joim.13634 37004213

[12] Karsdal MA Tambiah J Felson D et al. Reflections from the OARSI 2022 clinical trials symposium: the pain of OA - deconstruction of pain and patient-reported outcome measures for the benefit of patients and clinical trial design Osteoarthritis Cartilage 2023 31 10 1293 1302 10.1016/j.joca.2023.06.006 37380011 PMC11184959

[13] Paskins Z Farmer CE Manning F et al. Research priorities to reduce the impact of musculoskeletal disorders: a priority setting exercise with the child health and nutrition research initiative method Lancet Rheumatol 2022 4 9 e635 e645 10.1016/S2665-9913(22)00136-9 36275038 PMC9584828

[14] No authors listed Developing healthcare products UK Research and Innovation (UKRI) 2023 https://www.ukri.org/councils/mrc/facilities-and-resources/find-an-mrc-facility-or-resource/mrc-regulatory-support-centre/developing-healthcare-products/experimental-medicine/#contents-list date last accessed 12 August 2024

[15] No authors listed Improvement Leaders’ Guide: Working with Groups, General Improvement Skills NHS Institute for Innovation and Improvement 2005 https://www.england.nhs.uk/improvement-hub/wp-content/uploads/sites/44/2017/11/ILG-1.3-Working-with-Groups.pdf date last accessed 31 July 2024

[16] Whittaker JL Runhaar J Bierma-Zeinstra S Roos EM A lifespan approach to osteoarthritis prevention Osteoarthritis Cartilage 2021 29 12 1638 1653 10.1016/j.joca.2021.06.015 34560260

[17] Beaudart C Lengelé L Leclercq V et al. Symptomatic efficacy of pharmacological treatments for knee osteoarthritis: a systematic review and a network meta-analysis with a 6-month time horizon Drugs 2020 80 18 1947 1959 10.1007/s40265-020-01423-8 33074440 PMC7716887

[18] St Clair CO Papadopoulos EJ Considerations in the assessment of clinical benefit with a focus on pain: a regulatory perspective Neurotherapeutics 2020 17 3 770 773 10.1007/s13311-020-00906-6 32779129 PMC7609809

[19] Culvenor AG Øiestad BE Hart HF Stefanik JJ Guermazi A Crossley KM Prevalence of knee osteoarthritis features on magnetic resonance imaging in asymptomatic uninjured adults: a systematic review and meta-analysis Br J Sports Med 2019 53 20 1268 1278 10.1136/bjsports-2018-099257 29886437 PMC6837253

[20] Bruyère O Cooper C Arden N et al. Can we identify patients with high risk of osteoarthritis progression who will respond to treatment? A focus on epidemiology and phenotype of osteoarthritis Drugs Aging 2015 32 3 179 187 10.1007/s40266-015-0243-3 25701074 PMC4366553

[21] Curtin F Schulz P Assessing the benefit: risk ratio of a drug--randomized and naturalistic evidence Dialogues Clin Neurosci 2011 13 2 183 190 10.31887/DCNS.2011.13.2/fcurtin 21842615 PMC3181998

[22] Whittaker JL Culvenor AG Juhl CB et al. OPTIKNEE 2022: consensus recommendations to optimise knee health after traumatic knee injury to prevent osteoarthritis Br J Sports Med 2022 56 24 1393 1405 10.1136/bjsports-2022-106299 36379676

[23] Lohmander LS Roemer FW Frobell RB Roos EM Treatment for acute anterior cruciate ligament tear in young active adults NEJM Evid 2023 2 8 EVIDoa2200287 10.1056/EVIDoa2200287 38320141

[24] Al-Omari B McMeekin P Patients’ preferences regarding osteoarthritis medications: an adaptive choice-based conjoint analysis study Patient Prefer Adherence 2020 14 2501 2515 10.2147/PPA.S283922 33376311 PMC7765685

[25] Parsons J Abel G Mounce LT Atherton H The changing face of missed appointments Br J Gen Pract 2023 73 728 134 135 10.3399/bjgp23X732249 36823055 PMC9976814

[26] Garriga C Goff M Paterson E et al. Clinical and molecular associations with outcomes at 2 years after acute knee injury: a longitudinal study in the Knee Injury Cohort at the Kennedy (KICK) Lancet Rheumatol 2021 3 9 e648 e658 10.1016/S2665-9913(21)00116-8 34476411 PMC8390381

[27] Voorberg WH Bekkers VJJM Tummers LG A systematic review of co-creation and co-production: embarking on the social innovation journey Public Management Review 2015 17 9 1333 1357 10.1080/14719037.2014.930505

[28] McMillan SS King M Tully MP How to use the nominal group and Delphi techniques Int J Clin Pharm 2016 38 3 655 662 10.1007/s11096-016-0257-x 26846316 PMC4909789

